# The Effects of School Holidays on Transmission of Varicella Zoster Virus, England and Wales, 1967–2008

**DOI:** 10.1371/journal.pone.0099762

**Published:** 2014-06-16

**Authors:** Charlotte Jackson, Punam Mangtani, Paul Fine, Emilia Vynnycky

**Affiliations:** 1 Department of Infectious Disease Epidemiology, London School of Hygiene and Tropical Medicine, London, United Kingdom; 2 Statistics, Modelling and Economics Department, Public Health England, London, United Kingdom; University of California Riverside, United States of America

## Abstract

**Background:**

Changes in children’s contact patterns between termtime and school holidays affect the transmission of several respiratory-spread infections. Transmission of varicella zoster virus (VZV), the causative agent of chickenpox, has also been linked to the school calendar in several settings, but temporal changes in the proportion of young children attending childcare centres may have influenced this relationship.

**Methods:**

We used two modelling methods (a simple difference equations model and a Time series Susceptible Infectious Recovered (TSIR) model) to estimate fortnightly values of a contact parameter (the per capita rate of effective contact between two specific individuals), using GP consultation data for chickenpox in England and Wales from 1967–2008.

**Results:**

The estimated contact parameters were 22–31% lower during the summer holiday than during termtime. The relationship between the contact parameter and the school calendar did not change markedly over the years analysed.

**Conclusions:**

In England and Wales, reductions in contact between children during the school summer holiday lead to a reduction in the transmission of VZV. These estimates are relevant for predicting how closing schools and nurseries may affect an outbreak of an emerging respiratory-spread pathogen.

## Introduction

Chickenpox is caused by varicella zoster virus (VZV), which is spread primarily by the respiratory route. In temperate regions, almost all individuals become infected with VZV during their lifetime, usually during childhood [1]. Several modelling studies have found that, similar to measles [2]–[6], VZV transmission is reduced during school holidays compared to termtime [5]–[7]. Two of these studies used historical data, from early 20^th^ Century Denmark [5] or from 1928–1973 in the USA [6]. A third study used a more recent, but relatively short, time series from France (1991–96) [7]. The importance of schools in the transmission of VZV is further supported by data from Massachusetts, USA (1952–61), which showed that 62% of cases were in children aged 5–9 years and 19% in children aged 6 years, i.e. having only recently started school [8].

Since the mid 1980s, the age distribution of GP (primary care) consultations for chickenpox in England and Wales has changed, with an increasing proportion of cases being aged <5 years and a decreasing proportion being aged 5–14 years [9]. This changing age distribution may be due to changes in contact patterns resulting from an increasing proportion of children attending preschool childcare centres [9]–[11]. This raises the question of how much increases in contact between pre-school children have reduced the influence of the school calendar on transmission of childhood infections. A similar change in the age distribution of reported chickenpox cases, attributed to increased urbanisation, has been reported for Massachusetts during 1942–51 compared with 1952–61, with an increasing proportion of cases being seen in children aged under 10 years [8].

In this paper, we apply two modelling approaches to long-term data (from 1967–2008) from England and Wales to estimate how the rate of VZV transmission changes during the year and whether its relationship to school holidays has changed over time. We calculate fortnightly values of a contact parameter, defined as the per capita rate of effective contact (i.e. contact sufficient to allow transmission) between two specific individuals [12]. The estimates indicate how school closure during an outbreak of a respiratory-spread pathogen might affect contact patterns and transmission of infection.

## Methods

### Datasets

#### Incidence data

The Royal College of General Practitioners (RCGP) Research and Surveillance Centre runs a sentinel primary care (GP) surveillance scheme in England and Wales [13]. Currently, data are collected electronically from ∼100 general practices in England and Wales (101 practices contributed data in 2009, covering a population of over 900 000 [14]). The RCGP provided weekly overall and age-specific GP consultation rates (1967–2008) for chickenpox (Figure S1 in File S1).

#### Birth rates

Fortnightly birth rates per 100,000, for 1967–2008, were estimated by dividing the annual number of births (available from the UK Office for National Statistics [15]) by 26 and using mid-year population estimates for England and Wales as the denominator [16]–[18].

#### School holiday dates

In England and Wales, school holiday dates are set locally by 326 Local Authority Districts (LADs). School holiday dates were identified from the websites of three randomly selected LADs from each of nine geographical regions [19]–[21]. Term dates were also available for the Inner London Education Authority (ILEA) for 1952/53 to 1979/80 (ILEA, unpublished data), and from the websites of the 13 LADs formed from the abolition of ILEA in 1990 [22]. On the basis of these data, fortnights 1, 7–8 (Easter, in April), 15–18 (late July to early September) and 26 (Christmas) were treated as holidays. Sensitivity analyses treated fortnights 1, 8, 16–18 and 26 as holidays.

### Modelling methods: overview

We used two approaches to explore how the rate of effective contact differs between termtime and holidays. Firstly, we used a simple mass action model, based on one used by Fine and Clarkson [2], [23], to calculate the contact parameter in each fortnight for individual years. In the second approach, we used “Time Series Susceptible Infectious Recovered” (TSIR) modelling [3], in which the contact parameter can vary seasonally, with a pattern assumed to be the same each year. Both methods are based on the following equations, which describe the number of susceptible (*S_t_*) and infectious (*I_t_*) individuals at each time point, *t*:
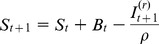
(1)

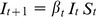
(2)

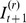
 represents the number of infections that are reported at time *t*+1; dividing this by the reporting fraction, *ρ* (the proportion of infections which are reported), provides an estimate of the total number of infectious individuals present in the population. *B_t_* represents the number of births which occurred at time *t*. The equations assume that individuals contact each other randomly. The time step used is the serial interval (the time between successive cases in a chain of transmission [24]), assumed to be two weeks [25].

### Estimating the reporting fraction

We used an approach from TSIR modelling to estimate the reporting fraction (the percentage of infections that are reported to the surveillance system) for both the simple mass action model and the TSIR model.

To estimate the reporting fraction (*ρ*), we first plotted the cumulative number of infections reported by a given time (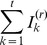) against the corresponding cumulative number of births (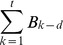), where *d* represents the duration of maternally derived immunity [3], assumed to be six months [26]. We then fitted a straight line through this plot and the reciprocal of the gradient of this line was taken to equal the reporting fraction. This method for estimating *ρ* follows from the observation [3] that, if all individuals become infected in their lifetime and infection is never fatal, Equation 1 can be rewritten as the equation of a straight line relating the cumulative number of births to the cumulative number of reported infections, with gradient 1/*ρ*:
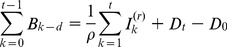
(3)
*D_t_* represents the residuals from the fitted line, corresponding to the difference between the mean number susceptible (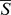) and the number susceptible at time *t* (i.e. *D_t_ = S_t_−* 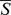) [3].

**Figure 1:**
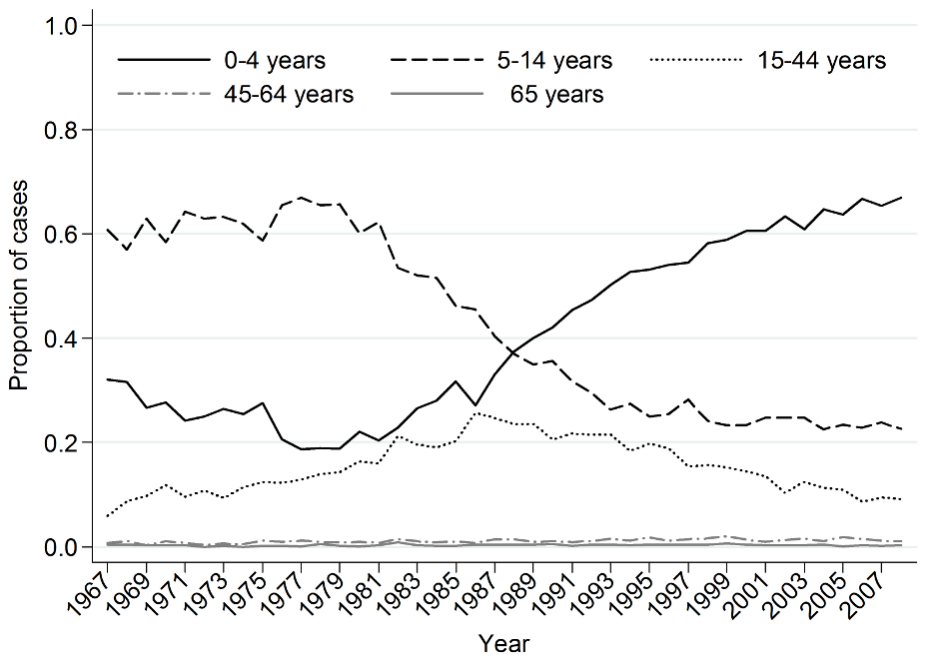
Proportion of chickenpox consultations in different age groups, 1967–2008.

As the age distribution of chickenpox consultations changed over time [9], [11], [27] (Figure 1) and symptom severity varies with age, we allowed the reporting fraction to differ between three periods defined by the age distribution of the consultations, i.e. we fitted separate lines to the plot of the cumulative number of cases against the cumulative number of births for the following periods: 1967–76 (most consultations were for 5–14 year-olds), 1977–97 (the proportion of consultations in 5–14 year-olds declined and that in 0–4 year-olds increased) and 1998–2008 (most consultations were for 0–4 year-olds).

### The simple mass action model

We used a modification of the method used by Fine and Clarkson [2] [23] to estimate fortnightly values of the contact parameter (*β_t_*) in each year using Equations 1 and 2. Equation 2 can be rearranged as follows in order to estimate fortnightly values of the contact parameter:
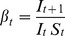
(4)


The values of *I_t_* and *I_t+1_* were taken from the RCGP data, corrected for under-reporting using the reporting fractions estimated from the TSIR model. The number of susceptible individuals at the start of 1967 was assumed to be either that estimated from the TSIR model (described below), the mean of the estimated values from the TSIR model when three time periods were considered separately (see below for further details), or 20% of the population (consistent with the observed proportion of the population that was found to be seronegative, according to serological data from England and Wales collected in 1996 [1]).

### TSIR model

#### Overview of TSIR modeling

The TSIR model [3], [5] is an extension of the simple mass action model in which Equation 1 is rearranged in order to estimate the reporting fraction as above, and a linear expression derived from Equation 2 is used to estimate fortnightly values of the contact parameter. Unlike the method used here with the simple mass action model, the TSIR method assumes that any seasonal patterns in the contact parameter are the same in all years analysed. Analyses of data using TSIR models often try to replicate the predictions that might be obtained in models which assume non-homogeneous mixing, by re-writing Equation 2 using an additional parameter, *α*, as:
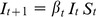
(2)
*α* is less than or equal to 1, with *α* =  1 corresponding to random mixing [3]. Although the interpretation of this parameter is not straightforward, its inclusion can help to correct inaccuracies resulting from modelling a continuous process using discrete time steps [28].

#### Estimating the contact parameters

The contact parameter *β_t_* for a given week *t* over different time periods (see above) and the parameter *α* were estimated by linear regression using Equation 5. This uses the estimated true numbers of chickenpox cases per 100,000 in fortnight *t* for *I_t_* (the reported number of cases per 100,000 corrected for under-reporting):
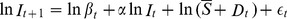
(5)This equation is obtained by substituting *S_t_ =* 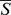 *+D_t_* into Equation 2 and taking logs of the resulting equation; *ε_t_* is an error term. Marginal profile likelihoods were used to estimate 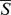
(see below) [5].

This regression was fitted separately for the periods 1967–76, 1977–97 and 1998–2008. In each regression, variations in *β_t_* during the course of the year were assumed to be the same in each year (e.g. the contact parameter in fortnight 1 of 1967, 1968, 1969,…1976 was assumed to be identical). The model was fitted repeatedly, each time using a different value of 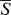 and estimating the associated values of *β_t_* and *α*. Goodness of fit was assessed using the log-likelihood deviance between the natural log of the estimated number of infections (the reported number corrected for under-reporting) and that predicted by Equation 5 as the goodness of fit criterion (see the Supplementary Information for the definition of the log-likelihood deviance). Values of 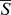 used in this fitting ranged from the minimum value which would allow 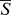 *+ D_t_* to be positive to 100,000 (Equation 5). The best-fitting values of ln(*β_t_*) and hence the contact parameter in fortnight *t* (*β_t_*) were taken from the model with the value of 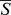 which resulted in the smallest log-likelihood deviance.

In sensitivity analyses we repeated the analyses after fixing the parameter *α* to equal 1 and used the Akaike’s Information Criterion (AIC) [29] to compare the resulting fit against that obtained without restricting the size of *α*. In addition, we explored the effect of assuming that the contact parameter followed the same cycle during the course of the year from 1967 to 2008.

#### Analyses of the consistency between model estimates and the observed data

We assessed the consistency between predictions based on the estimated values of *β_t_* and the observed data by comparing estimates of the number of cases each week (taken as the estimated reporting fraction multiplied by the exponents of the fitted values from the regression for each fortnight (Equation 5) (i.e. *I_t_*)) and those reported in the RCGP data using the correlation coefficient, *r*. Also, Equations 1 and 2 were evaluated using the estimates of *β_t_* and *α* to calculate the number of infectious cases each fortnight (per 100,000), which were compared against the observed data. The assumed number of infectious and susceptible individuals in 1967 were based on the reported chickenpox rate from the RCGP data (divided by the reporting fraction) and the estimated number susceptible.

### Relationship between the contact parameter and school holidays

For the estimates from the simple mass action and TSIR models, the percentage difference between *β_t_* during termtime and school holidays (all holidays or the summer holiday) was calculated using the expression 100 x ((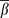
_term­_–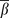
_holiday_)/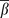
_term­_), where 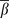 is the mean value of the contact parameter during the given time period. 95% CIs for the percentage difference were calculated using the bias-corrected and accelerated bootstrapping method [30].

All analyses were carried out in Stata, version 12.

## Results

From 1967–2008, chickenpox consultation rates were highest in 0–4 and 5–14 year-olds (Figure S1 in File S1). Consultation rates were highest in spring and lowest during the summer (typically reaching a minimum between weeks 37 and 39).

### Estimates of the contact parameters using the simple mass action model

**Figure 2:**
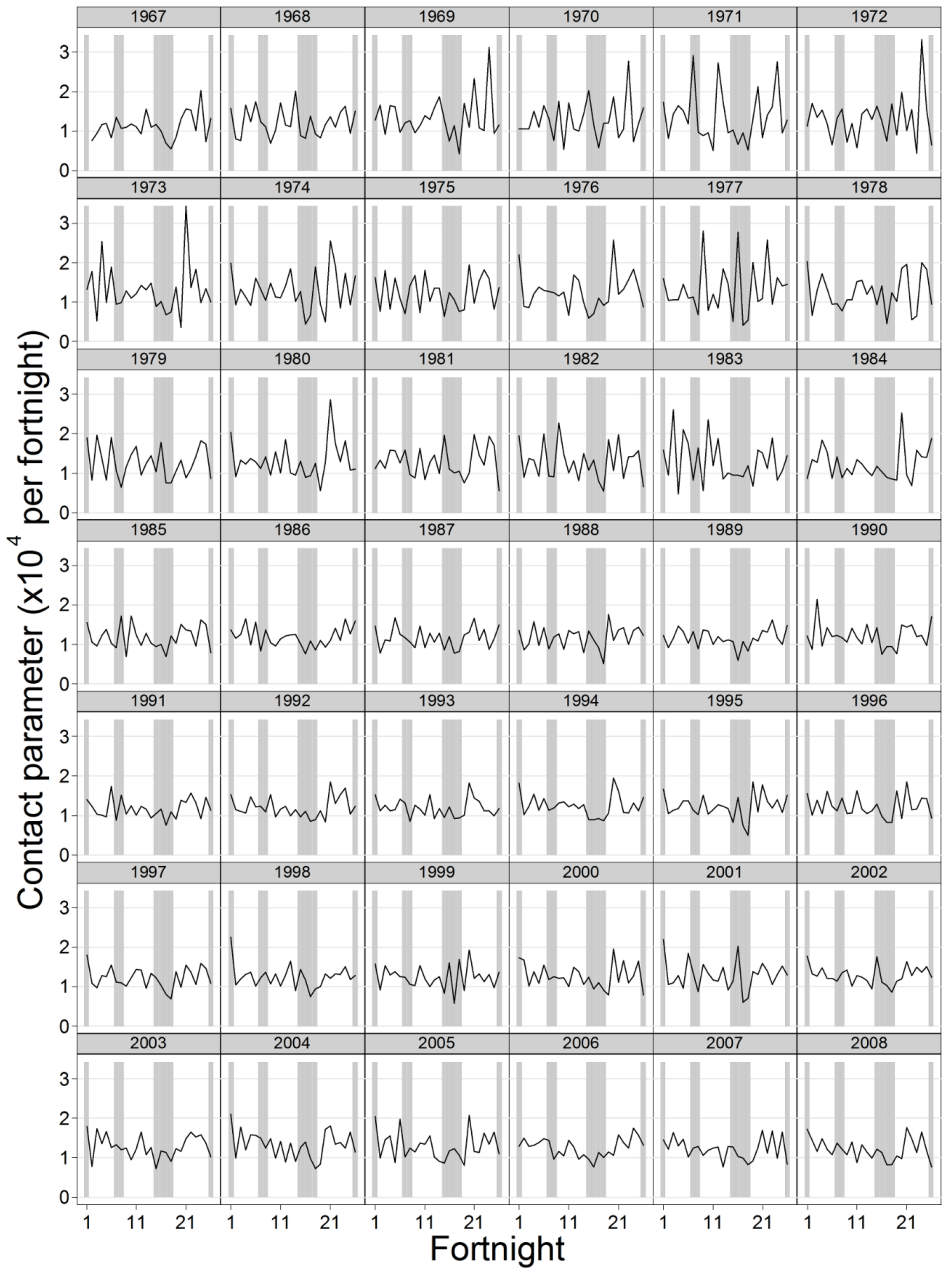
Fortnightly estimates of the contact parameter for chickenpox based on the simple mass action model. The proportion susceptible at the start of the time series was assumed to be 13%; shading shows approximate timing of school holidays.

**Figure 3:**
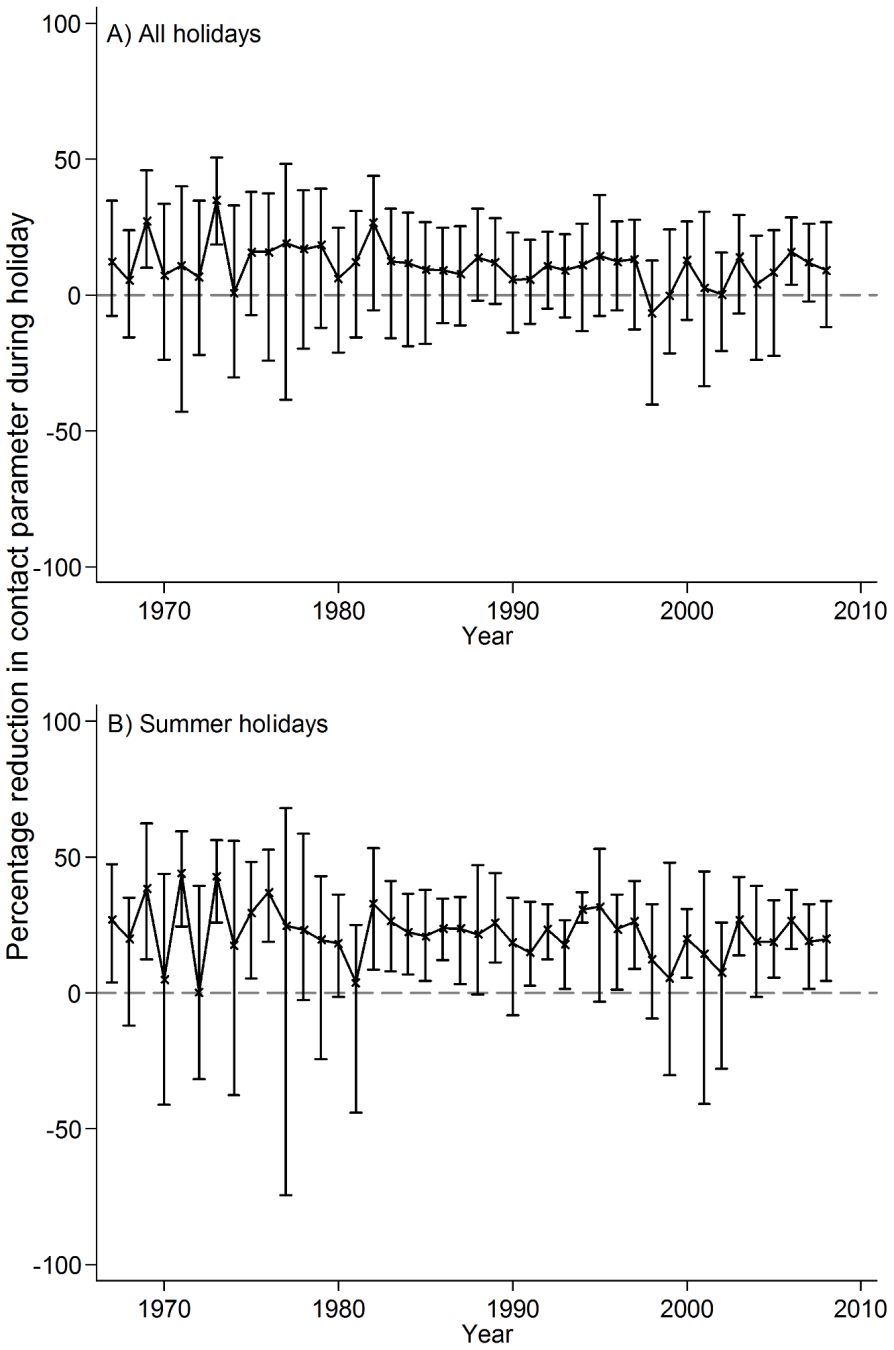
Percentage reduction in the contact parameter for chickenpox during school holidays, by year, 1967–2008. Estimates are based on the simple mass action model. Error bars show 95% confidence intervals.

The contact parameter estimated using the simple mass action model was in general lowest during the summer holiday, but seasonal patterns differed between years (Figure 2). Considering all school holidays across all years, the mean value of the contact parameter was 11% (95% CI −6–21%) lower during holidays than during termtime. Estimates for individual years ranged from a 30–35% reduction to a 7–8% increase. Most of the point estimates suggested that the contact parameter was lower during holidays than termtime, although the 95% CI included zero in most years (Figure 3A). Considering just the summer holidays, the mean estimate of the contact parameter during the holiday was 22% lower than that during termtime (95% CI 13–31%, Figure 3B). All of the point estimates for individual years indicated a reduction in the contact parameter during summer holidays, although this was sometimes small (ranging from 0.3% to 45%) and the CIs sometimes included zero (Figure 3B).

Estimates from the simple mass action model of the percentage difference between the contact parameter between termtime and school holiday were insensitive to the assumed number of susceptible individuals in 1967 and the dates of school holidays (Figures S10-12 in File S1).

### Analyses using TSIR modelling

The estimated reporting fraction was highest during 1977–98 (42.8%, 95% CI 42.7–42.9%), compared to 29.6% (95% CI 29.3–29.9%) and 31.6% (95% CI 31.4–31.8%) in 1967–76 and 1998–2008, respectively (Figure S2 in File S1).

Analyses of the RCGP data for the periods 1967–76, 1977–97 and 1998–2008, using Equation 5, suggested that on average 9% (95% CI 2–100%), 11% (95% CI 2–100%) and 18% (95% CI 1–100)%, respectively, of the population was susceptible to VZV, although the CIs were very wide. The corresponding estimates of *α* were 0.728 (95% CI 0.643–0.814), 0.682 (95% CI 0.619–0.744) and 0.704 (95% CI 0.617–0.791), respectively.

**Figure 4:**
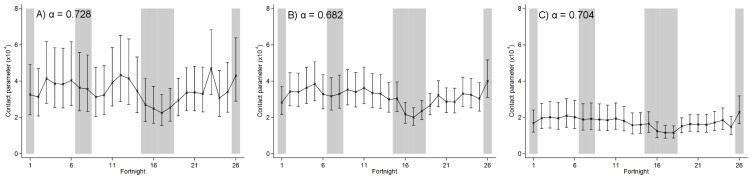
Fortnightly estimates of the contact parameter for chickenpox as estimated by TSIR modelling. A) 1967–76; B) 1977–97; C) 1998–2008; with values of α as shown. Error bars show 95% confidence intervals. Shaded rectangles show the approximate timing of school holidays. Fortnight 1 is the first two weeks of January; fortnight 26 is the last two weeks of December.

**Figure 5:**
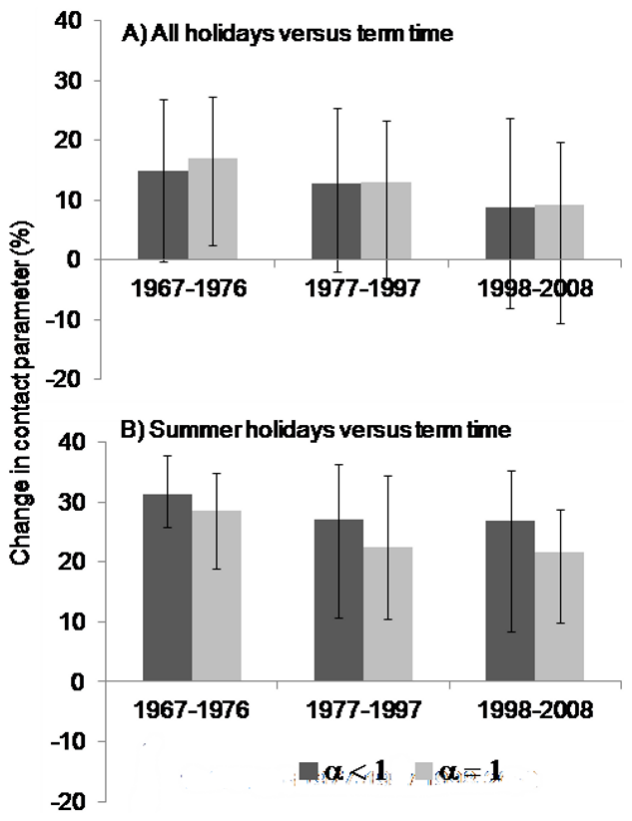
Estimated percentage difference between the contact parameter (per fortnight) for chickenpox during termtime and holidays. A) all school holidays; B) summer holidays, with different values of α. A positive value represents a reduction in the contact parameter during holidays.

Estimates of the contact parameter decreased over time, ranging from 2.25×10^−4^ to 4.71×10^−4^ per fortnight in 1967–76, 2.00×10^−4^ to 4.00×^−4^ per fortnight in 1977–97, and 1.15×10^−4^ to 2.30×10^−4^ per fortnight in 1998–2008 (Figure 4). The mean value of the contact parameter estimated for the summer holiday was 31% (95% CI 26–38%), 27% (95% CI 11–36%) and 27% (95% CI 8–35%) lower than that during termtime in 1967–76, 1977–97 and 1998–2008, respectively (Figures 4 and 5). All holidays considered together had no clear effect on the contact parameter. These estimates were fairly insensitive to the assumptions regarding the timing of holidays (Figure 5 and Figure S3 in File S1) and were similar to those obtained when *α* was constrained to equal 1 or when the TSIR model was applied to all the data from 1967–2008 (Figures S3–S9 in File S1).

**Figure 6:**
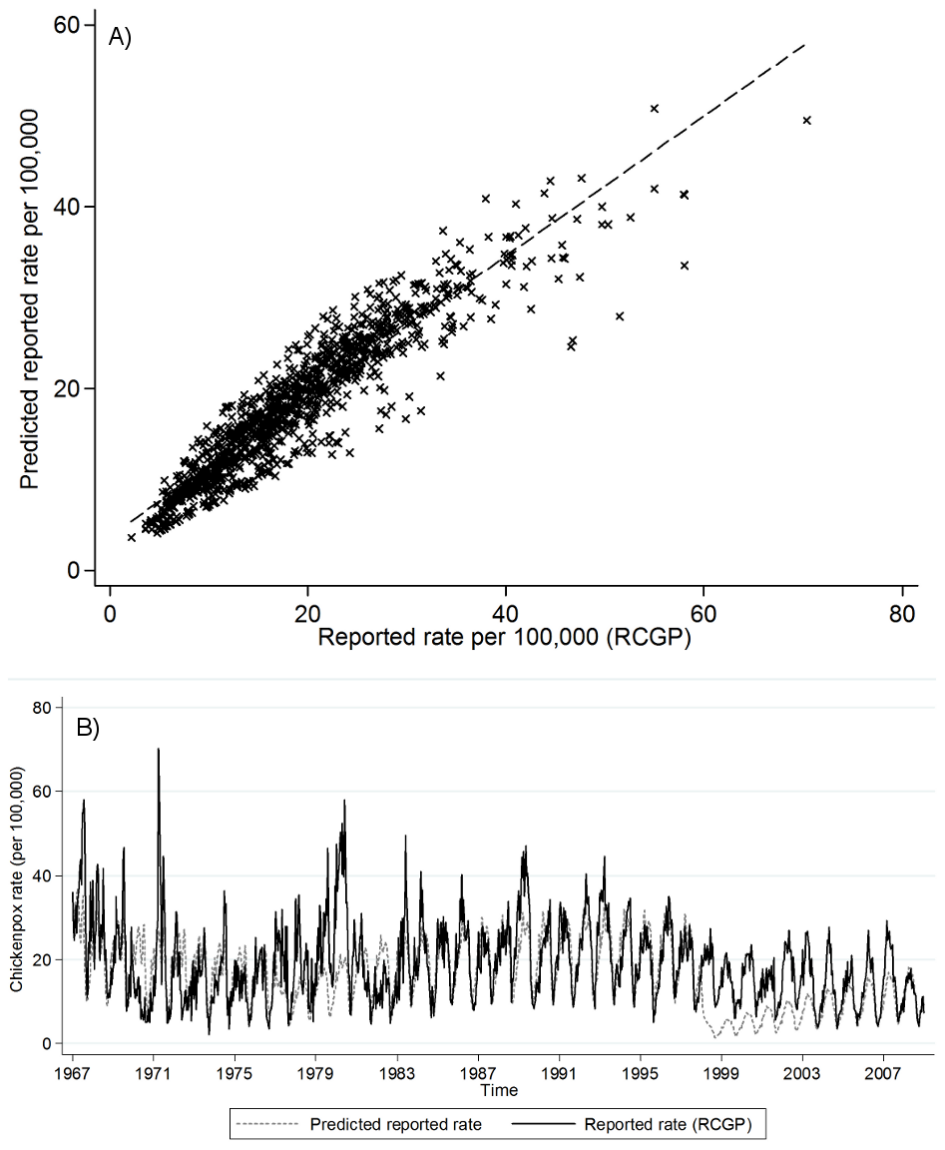
Assessment of model fit for chickenpox data, treating 1967–76, 1977–97 and 1998–2008 separately. A) Relationship between the RCGP chickenpox consultation rates and the fitted values from the regression (scaled down for under-reporting); B) RCGP data and the values predicted by the difference equations using the estimated contact parameters.

The fitted values of the number of chickenpox cases from the regression (scaled down for under-reporting) compared well with the observed RCGP data: the correlation coefficient was 0.929 (95% CI 0.920–0.937, Figure 6A). The output obtained after substituting the estimated contact parameters and *α* into the difference equations (Equations 1 and 2) also correlated reasonably well with the data (*r* = 0.631, 95% CI 0.594–0.666), although the correspondence was relatively poor during 1998–2003 (Figure 6B).

## Discussion

We estimate that contact between children sufficient for transmission of chickenpox is 22**–**31% lower during school summer holidays than during termtime. The effect of the summer holiday does not appear to have changed substantially over time, despite increases in the proportion of preschool children attending childcare [31]. The results based on the TSIR and simple mass action models were similar.

We used a long, contemporary time series to analyse the effects of school holidays on contact patterns, taking into account temporal changes in the reporting fraction. However, the comparisons of the contact parameter between termtime and holidays have some limitations. They do not account for other seasonal factors, such as weather, which might affect transmission (particularly temperature: several studies have found the highest incidence of chickenpox to occur in the coolest months of the year) [32]. Some fortnights might have been misclassified as holidays, due to slight geographic and temporal variations in holiday dates. The two-week time step used (consistent with the serial interval of chickenpox [25]) also introduces some error as some fortnights may comprise one week each of holiday and termtime. Finally, the analyses do not account for the possibility that children might be more likely to see a GP during termtime than during school holidays (e.g. if parents require advice regarding how long their child should be excluded from school). This would reduce the reporting fraction during holidays compared to termtime, acting to overestimate the effects of holidays on the contact parameter.

We did not formally assess the effects of the Christmas and Easter holidays on contact patterns, for several reasons. Firstly, consultation behaviour over the Christmas and New Year periods will differ from that during the rest of the year. Secondly, the Christmas holiday lasts only two weeks, meaning that any comparison of the *β_t_* estimates for termtime and the Christmas holiday from the TSIR model would be based on a single value for the holiday. Thirdly, the dates of the Easter holiday vary considerably between years, so the classification of fortnights around this period as holiday or termtime is uncertain.

The TSIR and simple mass action models are based on the same principles, and produced similar point estimates of the effects of school holidays on the transmission of VZV. The TSIR model is statistically rigorous, e.g. it easily allows calculation of confidence intervals for the weekly estimates of the contact parameter. However, it assumes that the annual pattern in the contact parameter (and therefore the effect of school holidays) is the same in all years. Our implementation of the simple mass action model allowed the seasonal pattern to differ between years but does not indicate the precision of the estimates of the fortnightly contact parameters. The absence of a clear trend in the effects of school holidays on the contact parameter as estimated using the simple mass action model therefore supports the conclusion from the TSIR model that there was no systematic change in the effects of school holidays over time.

Our results are consistent with previous studies of chickenpox. Monthly estimates of the contact parameter in New York City (1931**–**60) from an SIR model were lowest (∼65% of the mean value, i.e. a 35% reduction) during June and July [6]. The weekly contact parameter estimated from an SEIR model applied to data from France (1991**–**96) was ∼60% lower during the summer holiday than during a typical week in termtime [7]. The greater size of this reduction compared to our estimate may reflect differences in childcare practices, or in the length of the time period considered.

Monthly estimates of the basic reproduction number (R_0_, the average number of secondary infectious cases resulting from a single infectious person introduced into a completely susceptible population) for chickenpox, from a TSIR model applied to data from early 20^th^ Century Copenhagen, also appeared lowest during the summer holiday [5]. For example, R_0_ was ∼11**–**14 during June and July, and ∼25 in September.

Previous studies have suggested that the contact parameter for chickenpox between school-aged children has increased over time [10], which could result from increasing childcare attendance [31]. In contrast, our estimates from the TSIR model suggested that the contact parameter decreased over time. However, the absolute values of *β_t_* from the TSIR model are difficult to interpret, as they depend on the proportion of the population assumed to be susceptible at the start of the time period analysed. The point estimates of the average proportion susceptible increased over time, albeit with extremely wide CIs, which may explain the apparent decrease in the contact parameter. The results from the simple mass action model did not suggest a reduction in the contact parameter over time.

The average percentage of the population that is susceptible to chickenpox was estimated as 9%, 11% and 20% during 1967**–**76, 1977**–**97 and 1998**–**2008 respectively, but with considerable uncertainty. These point estimates are lower than published values of the proportion seronegative in the UK. Of 2091 specimens (taken from 1**–**20 year olds in England and Wales in 1996), 24% were seronegative [1]. Samples from 1**–**39 year olds from Sheffield taken between 1970 and 1992 varied in the proportion seronegative, from 42% (in 1974) to 22% (in 1992) [33]. However, the representativeness of these remainder blood samples from other laboratory tests is unclear, and the absence of age structure in the models makes it difficult to directly compare the estimates of the proportion susceptible with empirical data.

The seasonal pattern in the contact parameter was independent of the assumed proportion susceptible at the start of each time period, and remained consistent over time despite changes in preschool childcare attendance. This may be because preschool centres often (although not always) close over summer [34], which might augment the seasonal pattern compared to the situation where childcare attendance is limited. Increasing use of group childcare for school-aged children during holidays might reduce the influence of holidays on the contact parameter, so that overall the seasonal pattern remained relatively unchanged.

Our estimates of the reporting fraction (approximately 30%, 43% and 32% for 1967**–**76, 1977**–**97 and 1998**–**2008 respectively) are comparable to previous estimates from analyses of RCGP chickenpox data [35]–[37]. Previous analyses of the RCGP chickenpox data estimated the reporting fraction to be 36% by comparing consultation data from 1967**–**85 to serological data [35]. Other studies have also suggested a reporting fraction of 38% [36]. The age-specific true numbers of chickenpox cases occurring between 1991 and 2000 have been estimated by calculating the age-specific force of infection based on serological data [37]; comparing these estimated numbers of infections to the numbers of consultations in the RCGP data (reported in the same paper) produces an overall reporting fraction of 41% (43% for 0**–**4 year olds, 33% for 5**–**14 year olds, and 52% for 15**–**44 year olds). We were unable to estimate age-specific reporting fractions, but future work could extend Equation 3 to an appropriate multiple regression in order to estimate several reporting fractions. Temporal changes in the reporting fraction may reflect age-dependence in the propensity to consult a GP: the estimate reporting fraction and the proportion of consultations in 15**–**44 year-olds were both highest during 1977**–**97, and VZV infection is more severe in older age groups than in children [9], [37]. Other authors have fitted more complex functions, such as splines, to estimate the reporting fraction for other childhood infections, rather than the linear regressions which we used (Equation 3) [38]. Although this would have allowed us to analyse the full period 1967**–**2008 whilst still accounting for changes in the reporting fraction over time, we chose to use the linear regressions for simplicity and because the data for each time period were well approximated by a straight line (Figure S2 in File S1).

An alternative method of estimating the reporting fraction involves comparing age-specific serological data with the cumulative proportion of a birth cohort reported to have had the infection by a given age, as done for measles [2]. This would less easily allow the investigation of changes in the reporting fraction over time, as repeat serological surveys would be needed. Age-specific seroprevalence data based on sera taken between 1966 and 1992 in Sheffield, UK, have been published [33] but the relatively small sample size in some age groups and the unclear generalisability to the wider UK population may limit the reliability with which these data could be used to assess temporal changes in the reporting fraction.

Contact surveys (in which participants report the number of individuals with whom they make contact) [39] provide an alternative method of measuring the effects of school holidays on contact patterns. For example, children reported 19% [40] or 50% [41] fewer daily contacts during school holidays than termtime in Belgium and England, respectively. Results from modelling studies, such as ours, provide additional evidence to complement these estimates, and refer specifically to effective contact (the nature of which may be difficult to define in contact surveys).

Transmission of other viral infections spread by the respiratory route, including measles [2] [5], [6], mumps [5], [6] and influenza [42], appears to be reduced during school holidays to an extent similar to that which we estimated for chickenpox [2], [6], [42]. For example, the contact parameter for measles in England and Wales (pre-vaccination) was approximately 27% lower during holidays than termtime [2], despite the fact that measles is considered to be more transmissible than VZV [43]. Influenza surveillance data from France (1984**–**2006) suggest a 20**–**29% reduction in transmission between children during school holidays [42]. Transmission is likely to be affected by school closures only if they last longer than the serial interval of the given infection [9].

School closures, with or without concurrent closure of nurseries, may be considered as an outbreak control measure [44], [45], particularly if pharmaceutical interventions are unavailable. Our estimates of the effect of school holidays on transmission of chickenpox are indicative of how such closures might influence effective contact patterns for a respiratory-spread infection.

## Supporting Information

File S1 **Supporting information, figures and tables.** This file contains: Calculation of the deviance, Alternative TSIR modelling analyses, Additional analyses using the simple mass action model, and Figure S1-Figure S12. Figure S1, Weekly overall and age-specific GP consultation rates for chickenpox, England and Wales 1967–2008 (RCGP). Figure S2, Plot of the cumulative birth rate per 100,000 against the cumulative reported rate of chickenpox per 100,000, 1967–2008. Figure S3, Estimated percentage difference between the contact parameter (per fortnight) for chickenpox during termtime and that during A) all school holidays (fortnights 1, 8, 16–18 and 26) and B) summer holidays (fortnights 16–18), as estimated from the TSIR model. Figure S4, Fortnightly estimates of the contact parameter for chickenpox for 1967–76 (column 1), 1977–97 (column 2) and 1998–2008 (column 3), assuming *α = *1. Figure S5, Assessment of model fit for chickenpox data, assuming that *α = *1. A) Relationship between the RCGP chickenpox consultation rates and the fitted values from the regression for the periods 1967–76, 1977–1997 and 1998–2008 separately; B) Time series of RCGP data and predictions from difference equations for the periods 1967–76, 1977–97 and 1998–2008 separately; C) and D) as A) and B) but based on the full period 1967–2008. Figure S6, Fortnightly estimates of the contact parameter for chickenpox (1967–2008), for *α = *0.717. Figure S7, Assessment of model fit for chickenpox data, using the full period 1967–2008 and estimating *α* as 0.717. A) Relationship between the RCGP chickenpox consultation rates and the fitted values from the regression; B) Relationship between the RCGP data and the values predicted by the difference equations using the estimated contact parameters. Figure S8, Estimated percentage reductions in the contact parameter (per fortnight) for chickenpox during school holidays, based on the full period 1967–2008. Figure S9, Fortnightly estimates of the contact parameter for chickenpox, based on the full time period 1967–2008 and assuming *α = *1. Figure S10, Fortnightly estimates of the contact parameter for chickenpox based on the simple mass action model and RCGP consultation data, assuming that 20% of the population was susceptible to infection at the beginning of the time series. Figure S11, Fortnightly estimates of the contact parameter for chickenpox based on the simple mass action model and RCGP consultation data and assuming the proportion susceptible at the start of the time series to be 12% (the mean of the mean percentage susceptible during 1967–76, 1977–97 and 1998–2008); 13%. Figure S12, Percentage reduction in the contact parameter for chickenpox during school holidays, by year, 1967–2008 (simple mass action model), based on alternative assumptions about the dates of school holidays.
(DOCX)
