# Supplementary material for: The Effects of School Holidays on Transmission of Varicella Zoster Virus, England and Wales, 1967–2008
Source: PLoS One. 2014 Jun 16;9(6):e99762. doi: 10.1371/journal.pone.0099762 (PMC4059708; doi:10.1371/journal.pone.0099762)
Supplement: File S1 — Supporting information, figures and tables. This file contains: Calculation of the deviance, Alternative TSIR modelling analyses, Additional analyses using the simple mass action model, and Figure S1-Figure S12. Figure S1, Weekly overall and age-specific GP consultation rates for chickenpox, England and Wales 1967–2008 (RCGP). Figure S2, Plot of the cumulative birth rate per 100,000 against the cumulative reported rate of chickenpox per 100,000, 1967–2008. Figure S3, Estimated percentage difference between the contact parameter (per fortnight) for chickenpox during termtime and that during A) all school holidays (fortnights 1, 8, 16–18 and 26) and B) summer holidays (fortnights 16–18), as estimated from the TSIR model. Figure S4, Fortnightly estimates of the contact parameter for chickenpox for 1967–76 (column 1), 1977–97 (column 2) and 1998–2008 (column 3), assuming α = 1. Figure S5, Assessment of model fit for chickenpox data, assuming that α = 1. A) Relationship between the RCGP chickenpox consultation rates and the fitted values from the regression for the periods 1967–76, 1977–1997 and 1998–2008 separately; B) Time series of RCGP data and predictions from difference equations for the periods 1967–76, 1977–97 and 1998–2008 separately; C) and D) as A) and B) but based on the full period 1967–2008. Figure S6, Fortnightly estimates of the contact parameter for chickenpox (1967–2008), for α = 0.717. Figure S7, Assessment of model fit for chickenpox data, using the full period 1967–2008 and estimating α as 0.717. A) Relationship between the RCGP chickenpox consultation rates and the fitted values from the regression; B) Relationship between the RCGP data and the values predicted by the difference equations using the estimated contact parameters. Figure S8, Estimated percentage reductions in the contact parameter (per fortnight) for chickenpox during school holidays, based on the full period 1967–2008. Figure S9, Fortnightly estimates of the contact parameter for chic [file pone.0099762.s001.docx]

## **THE EFFECTS OF SCHOOL HOLIDAYS ON TRANSMISSION OF VARICELLA ZOSTER VIRUS, ENGLAND AND WALES, 1967-2008: SUPPORTING INFORMATION**

**Calculation of the deviance**

For each TSIR model, the deviance (D) was calculated using the following expression:

$$D=2(L_{s}- L_{f})$$

where *L_s_* and *L_f_* are the log-likelihoods of the saturated model and_­_ the fitted model, respectively, based on the normal distribution.

**Alternative TSIR modelling analyses**

*Analyses treating the data as three series and assuming that* α *= 1*

When the three periods 1967-76, 1977-97 and 1998-2008 were analysed separately and *α* was assumed to equal 1, $\bar{S}$ was estimated as ~12,000 (12%, 95% CI 2-100%), ~14,000 (14%, 95% CI 3-100%) and ~20,000 (20%, 95% CI 1.5-100%) for the three periods. The mean value of the contact parameter was estimated to be 17% (95% CI 2-27%), 13% (95% CI -3-23%) and 9% (95% CI -11-20%) lower during school holidays than during term time during 1967-76, 1977-97 and 1998-2008, respectively (Figures S3 and S4). The corresponding values for the summer holidays were 29% (95% CI 19-35%), 22% (95% CI 11-34%) and 22% (95% CI 10-29%).

The correlation coefficient between the fitted values from the regression and the RCGP data was 0.955 (95% CI 0.950 – 0.960, Figure S5A). When the estimates of *β*_t_ were substituted into the difference equations (Equations 1 and 2) and the equations were evaluated to calculate the numbers of infectious and susceptible individuals per 100,000, with *α* = 1, the results failed to reproduce the RCGP data (*r* = 0.254, 95% CI 0.198 – 0.309, Figure S5B).

*Analyses of the full series (1967-2008), including estimation of* α

When the data were treated as one series and *α* was estimated as 0.717, $\bar{S}$ was estimated as approximately 10200 (~10% susceptible in a population of 100,000, 95% CI 3-100%). With the respective best-fit estimates of $\bar{S}$, the model which did not assume that *α* = 1 gave a better fit to the data than that which assumed *α* = 1 (for the full series, AIC was 276 for *α* = 1 and 108 when *α* was estimated as 0.717).

With *α* = 0.717, the estimates of *β_t_* ranged from 1.9x10^-4^ (95% CI 1.6 x 10^-4^ – 2.2 x 10^-4^) to 3.7x10^-4^ (95% CI 3.1x 10^-4^ – 4.4 x 10^-4^) per fortnight (Figure S6). The minimum occurred in fortnight 17 (early August) and the maximum in fortnight 26. The second highest estimate was 3.5x10^-4^ (95% CI 2.9 x 10^-4^ – 4.2 x 10^-4^) per fortnight (fortnight 5, early March).

The estimated values of the chickenpox consultation rate derived from the regression model correlated well with the RCGP data (*r* = 0.936, 95% CI 0.928 – 0.943, Figure S7A). The predictions from the difference equations also correlated reasonably well with the RCGP data, with *r* = 0.702 (95% CI 0.671 – 0.731), although the difference equations tended to underestimate the RCGP rates when these were high (Figure S7B). This contrasts with the model which assumed *α* = 1, which did not reproduce the annual cycles in incidence (see below).

The contact parameter was 12% (95% CI -3-24%) lower during all holidays combined than during term time. There was evidence of a reduction in the contact parameter of 28% (95% CI 14-35%) during the summer holiday compared to term time (Figure S8).

*Analyses treating the data as a single series and assuming that* α *= 1*

When the full time series was analysed with the assumption that *α* = 1, $\bar{S}$ was estimated as 13000 (i.e. 13% susceptible in a population of 100 000, 95% CI 3-100%). The estimates of *β_t_* ranged from 5.4x10^-5^ (95% CI 5.0 x 10^-4^ – 5.9 x 10^-4^) to 10.2x10^-5^ (95% CI 9.4 x 10^-4^ – 11.1 x 10^-4^) per fortnight (Figure S9). The estimated contact parameter was 13% (95% CI -4-22%) lower during school holidays than term time, or 24% (95% CI 15-35%) lower during summer holidays than term time (Figure S8).

The fitted values of the consultation rates from the regression with *α* = 1 correlated well with the RCGP data (Figure S5C), with a correlation coefficient (*r*) of 0.965 (95% CI 0.961 – 0.969). When the estimates of $\bar{S}$ and *β_t_* were substituted into the difference equations (Equations 1 and 2), the predicted consultation rates were not consistent with the RCGP data, failing to reproduce the annual outbreaks (Figure S5D). The estimates derived without the assumption that *α* = 1 were much more consistent with the data (Figure S7).

*Analyses treating the data as three series using alternative assumptions about school holiday dates*

Assuming that school holidays occurred in fortnights 1, 8, 16-18 and 26, the estimated effects of the school holidays on the contact parameter were similar to those obtained in the main analysis (Figure S3). There was evidence that the contact parameter was lower during summer holidays than during term time, but not of a similar relationship between the contact parameter and all holidays considered together.

When *α* was not constrained to equal 1, the contact parameter was estimated to be 15% (95% CI -5 – 30%), 15% (95% CI -7 – 29%), and 11% (95% CI -12 – 28%) lower during school holidays than during term time in 1967-76, 1977-97 and 1998-2008, respectively. Assuming that *α* = 1, the corresponding estimates were 13% (95% CI -8 – 25%), 12% (95% CI -9 – 24%) and 9% (95% CI -20 – 22%).

The contact parameter was estimated to be 32% (95% CI 27-38%), 33% (95% CI 27-39%) and 34% (95% CI 28-37%) lower during summer holidays than during term time in 1967-76, 1977-97 and 1998-2008, respectively, when *α* was estimated. When *α* was assumed to equal 1, the corresponding estimates were 26% (95% CI 13-34%), 25% (95% CI 10-35%) and 26% (95% CI 21-30%).

*Analyses treating the data as a single series using alternative assumptions about school holiday dates*

Alternative assumptions about school holiday dates had little effect on the estimates of the effect of school holidays on the contact parameter based on the full time series (Figure S8). When *α* was not assumed to equal 1, the contact parameter was estimated to be 14% (95% CI -9-28%) lower during school holidays than during term time, or 33% (95% CI 29-37%) lower during summer holidays. The corresponding estimates with the assumption that *α* = 1 were 12% (95% CI -12-33%) and 25% (95% CI 14-33%).

**Additional analyses using the simple mass action model**

The relationship between the contact parameter and school holidays, assuming that either 20% or 12% (the mean of the three values of $\bar{S}$ ) of the population was susceptible to infection at the start of the time series, was similar to that assuming that 13% were susceptible (Figures S10 and S11). As in the TSIR analyses, the precise assumptions about the dates of school holidays had little effect on the estimates of the relationship between the contact parameter and school holidays (Figure S12).

**Supporting Figures and Tables**

*Figure S1. Weekly overall and age-specific GP consultation rates for chickenpox, England and Wales 1967-2008 (RCGP). Note the differing scales on the y-axes.*

**

*Figure S2. Plot of the cumulative birth rate per 100,000 against the cumulative reported rate of chickenpox per 100,000, 1967-2008. Births are lagged by six months to allow for maternally derived immunity. The solid black line shows the data; the dashed lines are three straight lines fitted to the data for the periods 1967-76 (red), 1977-97 (grey) and 1998-2008 (orange).*

**

*Figure S3. Estimated percentage difference between the contact parameter (per fortnight) for chickenpox during termtime and that during A) all school holidays (fortnights 1, 8, 16-18 and 26) and B) summer holidays (fortnights 16-18), as estimated from the TSIR model. A positive value represents a reduction in the contact parameter during holidays.*

*Figure S4. Fortnightly estimates of the contact parameter for chickenpox for 1967-76 (column 1), 1977-97 (column 2) and 1998-2008 (column 3), assuming α = 1. Error bars show 95% confidence intervals. Shaded rectangles show the approximate timing of school holidays. Fortnight 1 is the first two weeks of January; fortnight 26 is the last two weeks of December.*

| ** | ** | ** |
| --- | --- | --- |

*Figure S5. Assessment of model fit for chickenpox data, assuming that α = 1. A) Relationship between the RCGP chickenpox consultation rates and the fitted values from the regression for the periods 1967-76, 1977-1997 and 1998-2008 separately; B) Time series of RCGP data and predictions from difference equations for the periods 1967-76, 1977-97 and 1998-2008 separately; C) and D) as A) and B) but based on the full period 1967-2008.*

|   A |   B |
| --- | --- |
| **  C | **  D |

*Figure S6. Fortnightly estimates of the contact parameter for chickenpox (1967-2008), for α = 0.717. Error bars show 95% confidence intervals. Shaded rectangles show the approximate timing of school holidays. Fortnight 1 is the first two weeks of January; fortnight 26 is the last two weeks of December.*

**

*Figure S7. Assessment of model fit for chickenpox data, using the full period 1967-2008 and estimating* α *as 0.717. A) Relationship between the RCGP chickenpox consultation rates and the fitted values from the regression; B) Relationship between the RCGP data and the values predicted by the difference equations using the estimated contact parameters.*

| **  A | **  B |
| --- | --- |

*Figure S8. Estimated percentage reductions in the contact parameter (per fortnight) for chickenpox during school holidays, based on the full period 1967-2008. Holidays were assumed to occur in fortnights 1, 7, 8, 15-18 and 26 or in fortnights 1, 8, 16-18 and 26 (“alternative” dates). Dark grey: α = 1; light grey: α = 0.717. Error bars show 95% confidence intervals.*

*Figure S9. Fortnightly estimates of the contact parameter for chickenpox, based on the full time period 1967-2008 and assuming α = 1. Error bars show 95% confidence intervals. Shaded rectangles show the approximate timing of school holidays.*

**

*Figure S10. Fortnightly estimates of the contact parameter for chickenpox based on the simple mass action model and RCGP consultation data, assuming that 20% of the population was susceptible to infection at the beginning of the time series. Shading shows approximate timing of school holidays.*

**

*Figure S11. Fortnightly estimates of the contact parameter for chickenpox based on the simple mass action model and RCGP consultation data and assuming the proportion susceptible at the start of the time series to be 12% (the mean of the mean percentage susceptible during 1967-76, 1977-97 and 1998-2008); 13%; shading shows approximate timing of school holidays.*

**

*Figure S12. Percentage reduction in the contact parameter for chickenpox during school holidays, by year, 1967-2008 (simple mass action model), based on alternative assumptions about the dates of school holidays. Error bars show 95% confidence intervals.*

**
